# Expressions of MMP-12, TIMP-4, and Neutrophil Elastase in PBMCs and Exhaled Breath Condensate in Patients with COPD and Their Relationships with Disease Severity and Acute Exacerbations

**DOI:** 10.1155/2019/7142438

**Published:** 2019-04-17

**Authors:** Wendong Hao, Manxiang Li, Yunqing Zhang, Cailian Zhang, Yani Xue

**Affiliations:** ^1^Department of Respiratory and Critical Care Medicine, The Affiliated Hospital of Yan'an University, Yan'an, 716099 Shaanxi, China; ^2^Department of Respiratory and Critical Care Medicine, The First Affiliated Hospital of Xi'an Jiaotong University, Xi'an, 710061 Shaanxi, China

## Abstract

**Objective:**

The purpose of this study was to compare matrix metalloproteinase-12 (MMP-12), neutrophil elastase (NE), and tissue inhibitor of metalloproteinase-4 (TIMP-4) in peripheral blood of patients with chronic obstructive pulmonary disease (COPD) and controls. At the same time, MMP-12, NE, and TIMP-4 in exhaled breath condensate (EBC) were also evaluated.

**Methods:**

Peripheral blood and EBC samples from COPD patients and healthy controls were collected. In serum and EBC, MMP-12, NE, and TIMP-4 proteins were detected by enzyme-linked immunoassays. The mRNA expression levels of MMP-12, NE, and TIMP-4 in peripheral blood mononuclear cells (PBMCs) were analyzed by quantitative real-time polymerase chain reaction (qRT-PCR).

**Results:**

The concentration of TIMP-4 protein in EBC was lower in patients with COPD (*P* < 0.001). MMP-12 (*P* = 0.046), NE (*P* = 0.027), and TIMP-4 (*P* = 0.005) proteins in serum of patients with COPD showed higher levels of concentration. The mRNA of MMP-12 (*P* = 0.0067), NE (*P* = 0.0058), and TIMP-4 (*P* = 0.0006) in PBMCs of COPD patients showed higher expression levels. Compared with stable patients, mRNA expression level of NE (*P* = 0.033) in PBMCs of patients with acute exacerbation of COPD was increased. There were differences in the ratio of MMP-12/TIMP-4 in PBMC (*P* = 0.0055), serum (*P* = 0.0427), and EBC (*P* = 0.0035) samples between COPD patients and healthy controls. The mRNA expression of MMP-12 (*r* = −0.3958, *P* = 0.0186) and NE (*r* = −0.3694, *P* = 0.0290) in COPD patients was negatively correlated with pulmonary function. However, the mRNA expression of TIMP-4 (*r* = 0.2871, *P* = 0.0945) in PBMCs was not correlated with the FEV_1_ of the pulmonary function. Serum MMP-12 level was positively correlated with the MMP-12 level in EBC (*P* = 0.0387). The level of TIMP-4 in serum was not correlated with the level in the EBC sample (*P* = 0.4332).

**Conclusion:**

The expression levels of MMP-12, NE, and TIMP-4 in PBMCs and serum were elevated in COPD patients. In PBMCs of COPD patients, the mRNA expression level of NE may predict acute exacerbation, and MMP-12 mRNA expression level may be used to reflect the severity of airflow limitation. However, to better assess their diagnostic or prognostic value, larger studies are necessary.

## 1. Introduction

Chronic obstructive pulmonary disease (COPD) is a heterogeneous complex disease characterized by the progressive development of airflow limitation [1]. Protease/antiprotease imbalance, oxidative stress, cytokine hypothesis, immune imbalance, and infection hypothesis are considered to be the main pathogenesis of COPD [2], but the underlying mechanisms are still poorly understood, which has limited the development of new therapeutic and diagnostic approaches.

Matrix metalloproteinases (MMPs) are a family of similar proteins with similar structures that can degrade extracellular matrices, leading to structural damage and airway remodeling in COPD patients [3]. Their activity can be specifically inhibited by TIMPs, and the balance of MMPs/TIMPs plays a major role in maintaining normal tissue structure and physiological functions. Currently, there are many reports on MMP-8 and MMP-9, especially MMP-9, in patients with COPD, and the expression levels in serum [4], alveolar lavage fluid [5], and induced sputum [6] are significantly increased. However, with regard to MMP-12, there have been rather few reports on the mechanisms behind the destruction of lung tissue structure, airway remodeling, and emphysema formation in patients with COPD.

Compared with healthy controls, the concentration of MMP-12 in alveolar lavage fluid was significantly higher in COPD patients, which were closely related to the CT markers of small airway diseases and emphysema severity [7]. A study by Papakonstantinou et al. [8] showed that the gene expression of MMP-12 in bronchial tissues was negatively correlated with lung function FEV_1_. MMP-12 (interstitial lysin) has a wide range of hydrolysis substrates, such as type III, IV, and V collagen, laminin-1, gelatin, proteoglycans, and elastin. MMP-12 degrades extracellular matrices and elastin, increases production of cytokines and chemokines, and increases proteolysis by inactivation of protease inhibitors, ultimately leading to lung structural destruction and airway remodeling of COPD [9]. Neutrophil elastase (NE) is released by neutrophils and is an influential member of the serine protease family. NE not only degrades elastase but also stimulates the production and secretion of mucin, which eventually leads to high airway mucus secretion and airway obstruction in COPD patients [10]. NE has been demonstrated elevated in induced sputum and plasma of COPD patients [11].

The lungs are relatively difficult to sample, and commonly used technologies such as bronchoscopy tissue biopsy, bronchoalveolar lavage fluid, and percutaneous lung puncture are invasive. Exhaled breath condensate (EBC) is the latest technology for detecting the biochemical components of the respiratory tract, also known as biochemical lung function. It is used in the diagnosis and prognosis of lung diseases because it offers the advantages of being safe, noninvasive, simple to collect, and applicable for multiple repetitions [12]. Currently, a study by Sng et al. [13] showed elevated levels of MMP-9 and NE in the EBC of patients with COPD. However, our study has, for the first time, detected MMP-12 and TIMP-4 in the EBC of COPD patients. Given their crucial role in the pathophysiology of COPD, our study evaluated the utility of MMP-12 and TIMP-4 in the EBC of patients with COPD.

The aim of this study was to detect the levels of MMP-12, TIMP-4, and NE in serum, PBMCs, and EBC and to explore the role of these inflammatory mediators in the pathophysiology of COPD. Finally, we examined the concentrations of these mediators during acute exacerbations of COPD.

## 2. Materials and Methods

### 2.1. Study Design and Participants

The present study was conducted with the approval of the ethics committee of the Affiliated Hospital of Yan'an University, and written informed consent was provided by all the participating subjects. This study was conducted in accordance with the tenets of the Declaration of Helsinki.

COPD was diagnosed according to the Global Initiative for Chronic Obstructive Lung Disease (GOLD) guidelines [14]. Thirty-five participants with stable COPD or acute exacerbations were recruited for the study. Twenty-eight control subjects were selected from a group of healthy subjects who presented at the Department of Respiratory Medicine for regular health control or checkup. Detailed history was taken from all the participants. Acute exacerbation of COPD (AECOPD) was defined as an increase of at least two lower respiratory tract symptoms related to COPD (shortness of breath, sputum production (volume), sputum purulence, cough, wheezing, or chest tightness) or the new onset of two or more such symptoms, with at least one symptom lasting 3 or more days and for which antibiotics, systemic glucocorticoids, or both were prescribed.

Entry criteria for this clinical trial included having a ratio of forced expiratory volume in one second to forced vital capacity (FEV_1_/FVC) < 70%. COPD was staged in accordance with the GOLD guidelines: GOLD I (mild), FEV_1_ ≥ 80% predicted; GOLD II (moderate), FEV_1_ < 80% and ≥50% predicted; GOLD III (severe), FEV_1_ < 50% and ≥30% predicted; and GOLD IV (very severe), FEV_1_ < 30% predicted. The main exclusion criteria (for both patients and healthy controls) were as follows: malignant tumor, active tuberculosis, type 2 diabetes, autoimmune disease, inflammatory disease, heart failure, liver and kidney failure, cerebrovascular disease, inhaled/oral corticosteroid therapy, or lung disease (other than COPD for patients).

### 2.2. Levels of MMP-12, TIMP-4, and NE in EBC and Serum

Exhaled breath condensate (EBC) was collected using the condenser RTube (Respiratory Research Inc., USA). The aluminum cooling jacket was placed in a -80°C refrigerator and allowed to cool sufficiently to collect exhaled breath condensate. Before collection, all participants used distilled water to rinse the mouth and routinely wore a noseclip. During collection, subjects used a bite device and breathed through the bite device for 10-15 minutes. The collected EBC was transferred to a collection tube using a pipette and stored at -80°C.

MMP-12, TIMP-4, and NE levels in EBC and serum were analyzed by the ELISA technique using commercial kits (Human MMP-12, TIMP-4, and NE kits, R&D Systems, Wiesbaden-Nordenstadt, Germany) according to the manufacturer's instructions. The lower limits of detection for these assays are as follows: MMP-12, 0.156 ng/mL; TIMP-4, 0.08 ng/mL; and NE, 0.4 ng/mL. MMP-12, TIMP-4, and NE levels were determined for each sample in all subjects.

### 2.3. Isolation of PBMCs and Analysis of mRNA Expression

Peripheral blood samples from each subject were collected into vacutainer tubes containing ethylenediaminetetraacetic acid dipotassium salt as the anticoagulant. PBMCs were isolated from the whole blood samples within 2 hours of sample collection by Ficoll-Hypaque (Ficoll-Paque PLUS; GE Healthcare Bio-Sciences AB, Uppsala, Sweden) gradient centrifugation and immediately preserved in a −80°C freezer until the assay. Total RNA was extracted from the PBMCs in accordance with the manufacturer's instruction.

Quantitative real-time PCR for MMP-12, TIMP-4, and NE was performed using the human Sandwich High-Sensitivity ELISA kit (Boster Biological Technology, Wuhan, China) with QuantiTect Primer Assays (Wuhan Fast Test Technology Service Co. Ltd., Hubei, Qiagen) on the Applied Biosystems® 7500 Real-Time PCR Systems (Applied Biosystems Inc., Carlsbad, USA) under standard conditions with HPRT as the reference control.

### 2.4. Statistical Analysis

Data are expressed as median ± (range) or mean ± SD. Pearson's correlation coefficient was used for correlation studies. Data were analyzed using the D'Agostino and Pearson test for normality and the nonparametric Mann-Whitney *U* tests or parametric unpaired *t*-tests for statistical analysis. All data analyses were performed using GraphPad Prism 5 (GraphPad Software Inc., San Diego, CA, USA). Statistical significance was established with a *P* value < 0.05.

## 3. Results

### 3.1. Clinical Characteristics

This study included 10 patients with AECOPD, 25 patients with stable COPD, and 28 healthy controls. EBC and blood samples were collected from all participants for analysis. Compared with the healthy control group, patients in the COPD group were older, with a significant decrease in lung function FEV_1_ (*P* < 0.0001), FVC (*P* = 0.0008), FEV_1_/FVC (*P* < 0.0001), and DLco (*P* < 0.0001). In smoking history, smoking in COPD patients was significantly greater than that in healthy controls. In healthy controls, only 25% were ex-smokers, but current smokers/ex-smokers in COPD patients accounted for up to 91% (Table 1).

### 3.2. MMP-12 and TIMP-4 in EBC

**Figure 1:**
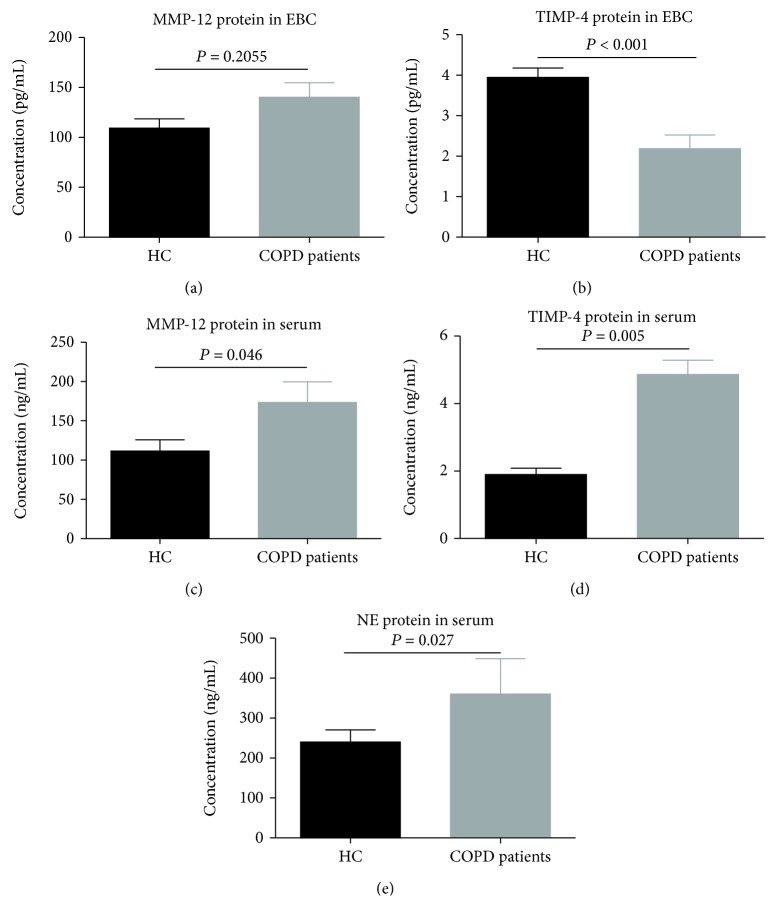
Expressions of protein for (a) MMP-12 and (b) TIMP-4 in EBC from patients with COPD (*n* = 35) and healthy controls (*n* = 28). Expression of protein for (c) MMP-12, (d) TIMP-4, and (e) NE in serum from patients with COPD (*n* = 35) and healthy controls (*n* = 28). All data are presented as mean ± SD.

EBC samples from all participants were tested. MMP-12 levels (95.5 ± 49.2 pg/mL) were increased in COPD patients compared with healthy controls (79.0 ± 37.6 pg/mL) (*P* = 0.2055, Figure 1(a)). The TIMP-4 level (1.2 ± 0.8 pg/mL) in EBC of patients with COPD was significantly lower than that of healthy controls (2.3 ± 1.1 pg/mL) (*P* < 0.001, Figure 1(b)).

### 3.3. MMP-12, TIMP-4, and NE in Serum

**Figure 2:**
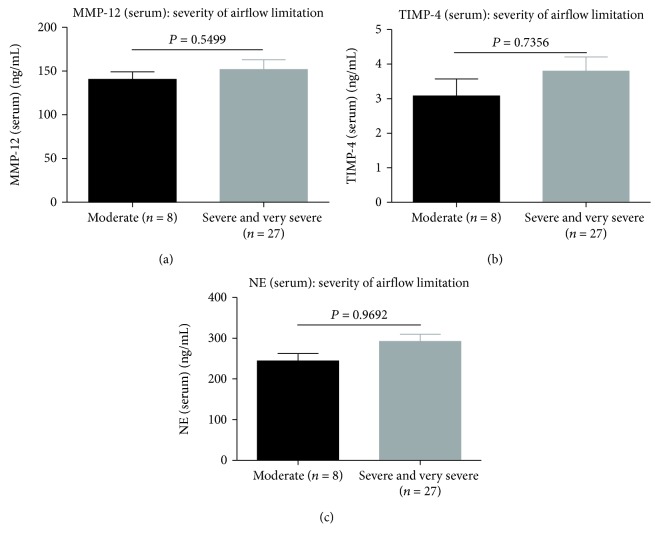
Expressions of serum protein levels for (a) MMP-12, (b) TIMP-4, and (c) NE between different severities of airflow limitation in COPD patients. All data are presented as mean ± SD.

Serum levels of MMP-12 (*P* = 0.046, Figure 1(c)), TIMP-4 (*P* = 0.005, Figure 1(d)), and NE (*P* = 0.027, Figure 1(e)) were higher in patients with COPD compared with healthy controls. Furthermore, serum concentrations of the above markers MMP-12, TIMP-4, and NE were not related to the stage of COPD and severity of airflow limitation. There were no significant differences in MMP-12, TIMP-4, and NE serum protein levels between different severity of airflow limitation in COPD patients (*P* = 0.5499, Figure 2(a); *P* = 0.7356, Figure 2(b); and *P* = 0.9692, Figure 2(c)).

### 3.4. Expression of mRNA for MMP-12, TIMP-4, and NE in PBMCs

**Figure 3:**
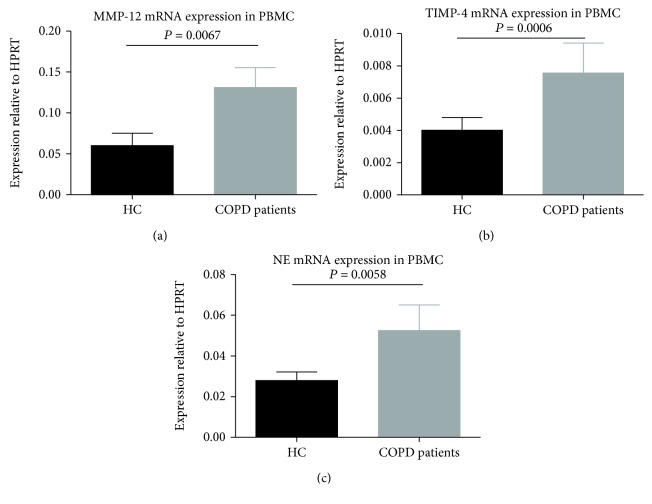
Expressions of mRNA for (a) MMP-12, (b) TIMP-4, and (c) NE in PBMCs from patients with COPD (*n* = 35) and healthy controls (*n* = 28). Data are presented as mean ± SD.

**Figure 4:**
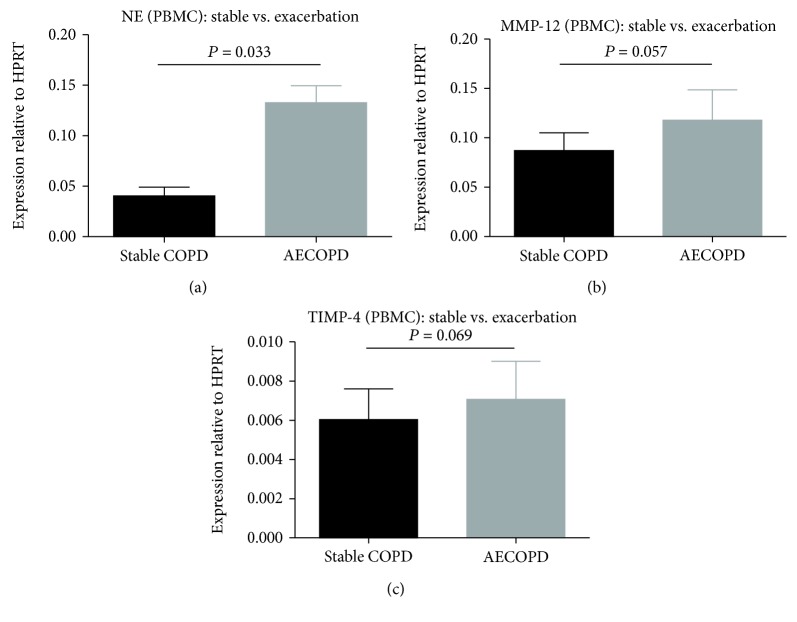
Expressions of mRNA for (a) NE, (b) MMP-12, and (c) TIMP-4 in PBMCs from patients with stable COPD (*n* = 22) and patients with AECOPD (*n* = 13). Data are presented as mean ± SD.

**Figure 5:**
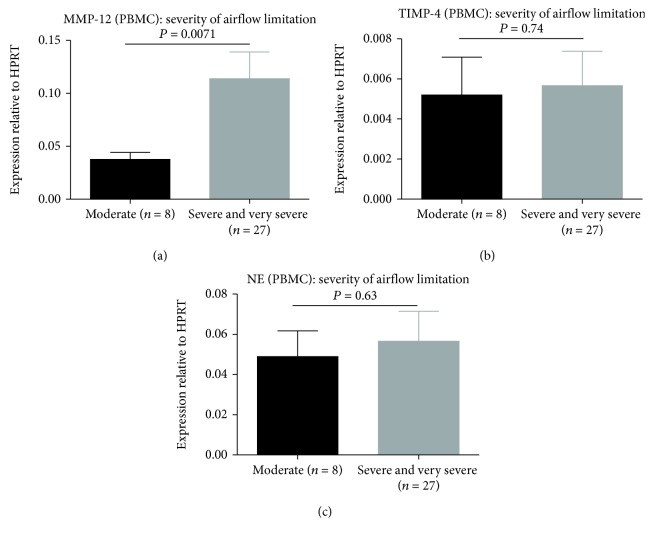
Expressions of mRNA for (a) MMP-12, (b) TIMP-4, and (c) NE in PBMCs from patients with COPD according to the severity of airflow limitation. Data are presented as mean ± SD.

The mRNA levels of MMP-12 (*P* = 0.0067; Figure 3(a)), TIMP-4 (*P* = 0.0006; Figure 3(b)), and NE (*P* = 0.0058; Figure 3(c)) in PBMCs of COPD patients were significantly higher than healthy controls. NE levels in patients with COPD who experienced an exacerbation were significantly elevated (*P* = 0.033, Figure 4(a)). MMP-12 and TIMP-4 mRNA levels were not significantly different between the two COPD subgroups (*P* = 0.057, Figure 4(b) and *P* = 0.069, Figure 4(c)). MMP-12 mRNA levels were markedly elevated in COPD patients (severe and very severe) according to the severity of airflow limitation (*P* = 0.0071, Figure 5(a)). However, there were no significant differences in TIMP-4 and NE mRNA expression levels between different severity of airflow limitation in COPD patients (*P* = 0.74, Figure 5(b) and *P* = 0.63, Figure 5(c)).

### 3.5. The Ratio of MMP-12/TIMP-4 in Different Samples

**Figure 6:**
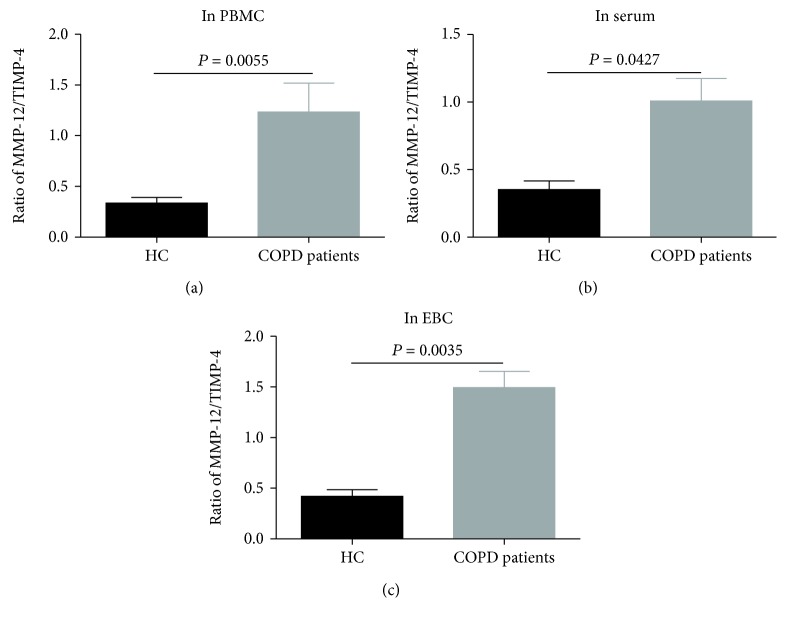
The ratios of MMP-12/TIMP-4 in (a) PBMC, (b) serum, and (c) EBC samples between COPD patients and healthy controls.

We examined the ratio of MMP-12/TIMP-4 in different samples of all participants. There were differences in the ratio of MMP-12/TIMP-4 in PBMCs (*P* = 0.0055, Figure 6(a)), serum (*P* = 0.0427, Figure 6(b)), and EBC (*P* = 0.0035, Figure 6(c)) samples between COPD patients and healthy controls.

### 3.6. Correlation Analysis between the Cytokines and Spirometry

**Figure 7:**
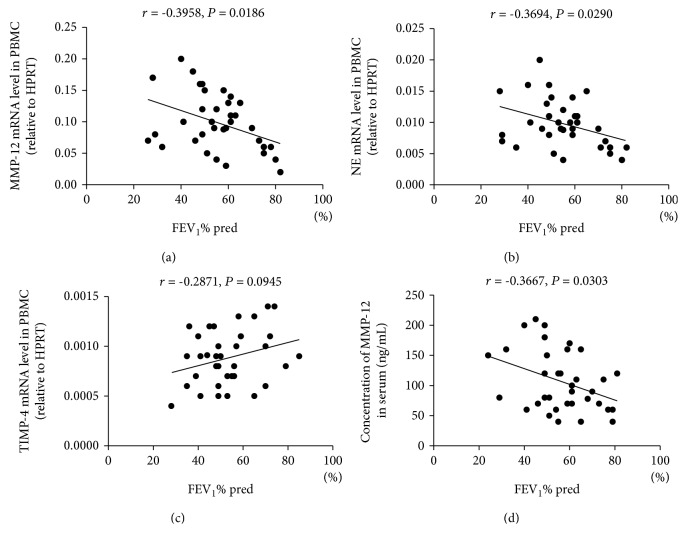
Correlations between lung function (expressed as FEV_1_% predicted) and mRNA expression of MMP-12, NE, and TIMP-4 in PBMCs and concentration of MMP-12 in serum from patients with COPD. (a) Correlation between FEV_1_ and expression of mRNA for MMP-12 in PBMCs. (b) Correlation between FEV_1_ and expression of mRNA for NE in PBMCs. (c) Correlation between FEV_1_ and expression of mRNA for TIMP-4 in PBMCs. (d) Correlation between FEV_1_ and concentration of MMP-12 in serum. Abbreviations: FEV_1_s, forced expiratory volume in 1 second.

MMP-12 mRNA levels were negatively correlated with pulmonary function FEV_1_%pred (*P* = 0.0186, Figure 7(a)). Levels of mRNA for NE were also negatively correlated with FEV_1_%pred (*P* = 0.0290, Figure 7(b)). However, TIMP-4 mRNA levels were not correlated with FEV_1_%pred (*P* = 0.0945, Figure 7(c)). Concentration of serum MMP-12 was also significantly negatively correlated with lung function FEV_1_%pred (*P* = 0.0303, Figure 7(d)).

### 3.7. Correlation of Inflammatory Mediator Levels in Serum and EBC Samples

**Figure 8:**
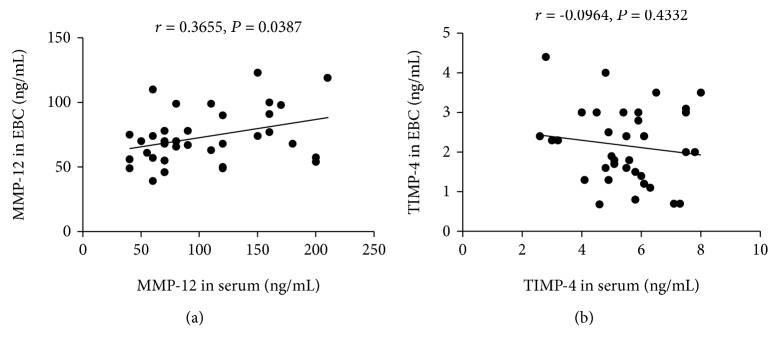
(a) Correlation between serum MMP-12 levels and MMP-12 levels in EBC. (b) Correlation analysis between serum TIMP-4 protein levels and TIMP-4 protein levels in EBC.

Serum MMP-12 levels were positively correlated with MMP-12 levels in EBC (*P* = 0.0387, Figure 8(a)). Although the concentration of TIPM-4 was decreased in EBC and increased in serum, the level of TIMP-4 in serum was not correlated with the level in the EBC sample (*P* = 0.4332, Figure 8(b)).

## 4. Discussion

The objective of our study was to compare MMP-12, TIMP-4, and NE in peripheral blood of patients with COPD and healthy donors. We measured the release of inflammatory biomarkers from PBMCs and EBC in order to investigate whether there were differences between the two subject groups. Finally, we examined the concentrations of these mediators during acute exacerbations of COPD.

Our study showed that expression levels of MMP-12 mRNA were significantly higher in PBMCs of COPD patients compared with nonsmoking controls. In addition, MMP-12 mRNA expression level was negatively correlated with the lung function FEV_1_%pred in patients with COPD, which is consistent with the results of Xu et al. [15]. Furthermore, the research revealed that the more severe was the airflow limitation in patients with COPD, the higher was the expression level of MMP-12 mRNA in PBMCs. However, one study has shown that there were no differences in the expression of MMP-12 between COPD patients and current smokers [16], suggesting that smoking itself may be an important factor influencing the expression of MMP-12 mRNA.

MMPs degrade various protein components in ECM, which play a pivotal role in tissue damage, remodeling, and repair associated with inflammation [17]. In patients with COPD, our study suggests that MMP-12 mRNA expression in PBMC and serum MMP-12 protein level was significantly elevated. This finding is in accordance with previous work [18], which indicated that serum MMP-12 levels were significantly higher in patients with COPD compared with the nonsmoking healthy control subjects. However, Imai et al. [19] pointed out that MMP-12 was not upregulated in smokers and MMP-12 mRNA could not be found in most normal lungs. Finlay et al. [20] cultured alveolar macrophages from emphysematous and control lungs, and although they found increases in the production of MMP-9, they found no differences in mRNA levels of MMP-2 and MMP-12 and no production of MMP-12 protein. Research by Gosselink et al. [21] has shown no association between MMP-12 gene expression and the severity of airflow limitation in COPD patients. Although the main function of MMPs is to break down the extracellular matrix and participate in numerous physiological and pathological processes in humans, it is difficult to interpret published literature reports because the exact role of MMPs may not be very evident in COPD. Moreover, the activity of MMPs is tightly regulated at both the level of gene transcription and protein translation. Chaudhuri et al. [22] reported that the concentration and activity of MMP-12 in induced sputum in patients with COPD were significantly higher than those in healthy controls, but there was no significant increase in plasma MMP-12 in patients with COPD. This report showed that the biological activity of MMPs in COPD is very complicated. Therefore, scholars must comprehensively evaluate a variety of factors in the study of emphysema and airway inflammation.

TIMP-4 differs from TIMP-1, -2, and -3 in its restricted expression pattern and its structure in the region of binding between TIMPs and MMPs. Therefore, it has been suggested that TIMP-4 has higher specificity for particular MMPs than TIMP-1, -2, and -3 [23]. Our data showed that TIMP-4 mRNA expression in PBMC and serum TIMP-4 protein level was significantly elevated. Furthermore, we have tried to detect the concentration of TIMP-4 protein in EBC of COPD patients. However, contrary to the expression levels in PBMCs and serum, TIMP-4 protein concentration decreased in EBC of patients with COPD. Navratilova et al. [24] had reported that TIMP-4 is significantly elevated in the serum of COPD patients. This is consistent with our findings. The study [24] also pointed out that TIMP-4 not only inhibits the proteolytic activity of MMP-1, -2, -3, -7, -8, and -9 but it is also considered to be a strong inhibitor of MMP-12, -14, -19, and -26. In COPD, TIMP-4 may contribute to the antiproteolytic activity of TIMP-1, -2, and -3 and may even inactivate MMPs that are not sufficiently inhibited by other TIMPs.

The imbalance of MMPs/TIMPs in the lungs has been implicated in the development of COPD or emphysema. Our study showed that there were considerable differences in the ratio of MMP-12/TIMP-4 in PBMC, serum, and EBC samples between COPD patients and healthy controls. Furthermore, TIMP-4 levels are increased in serum and decreased in EBC of COPD patients. It is well known that the detection techniques for samples such as serum, tissue, and cell culture are extremely mature. However, since the collection method and detection technology of EBC samples are not completely unified internationally [25], the value of EBC in the diagnosis, therapeutic evaluation, and prognosis of respiratory diseases is still in the exploratory stage [26]. We have for the first time sought to detect TIMP-4 protein levels in EBC. This finding might suggest that TIMP-4 plays an important role in the development of COPD. It is also necessary to further study the cell origin, MMP specificity, and role in the pathogenesis of COPD immunology.

NE is capable of degrading most components of the extracellular matrix of the lung and plays a crucial role in the lung destruction of emphysema. Our study has shown that NE mRNA expression was significantly higher in PBMCs of COPD patients compared with healthy controls. Moreover, NE mRNA expression was markedly increased in PBMCs of acute exacerbation of COPD compared to stable COPD. Chronic inflammation of the airways and lung tissue is a major pathological feature of COPD [2]. In the pathological process of airway remodeling and emphysema formation in COPD, macrophages, monocytes, neutrophils, other immune cells, neutrophil elastase, matrix metalloproteinases, interleukins and tumor necrosis factor-*α*, and other cytokines are involved [27]. Acute exacerbation of COPD is a deterioration of the condition that occurs on the basis of stable COPD. The principal cause of acute exacerbation is viral and/or bacterial infection, which leads to a marked increase in the inflammatory response of the airways and lung tissue [28]. A PBMC is mainly composed of lymphocytes and monocytes, so this may explain the increased mRNA expression of inflammatory mediators in AECOPD patients compared to patients with stable COPD. In our study, the biomarker NE in the circulation of COPD patients is significantly negatively correlated with FEV_1_, which is consistent with the study by Higashimoto et al. [29]. Similarly, one study has suggested that pulmonary function, CT scan, and the number of inflammatory cells in bronchoalveolar lavage (BAL) fluid were not indicators of COPD progression, but FEV_1_ was significantly decreased in subjects with elevated NE and alpha-1 antitrypsin in BAL fluid and plasma [30].

Our study has a few limitations. First, this study is a single-center study and the sample sizes of the healthy control group and the COPD patient group are relatively small. Second, patients with COPD are older, so it is sometimes difficult to find healthy age-matched controls. However, for this study, it is also challenging to recruit age-matched smoking controls without COPD. In addition, this study did not longitudinally compare the changes in the indicators before, during, and after the exacerbation of COPD. It was also recognized that phenotypic expression of chronic bronchitis and emphysema in COPD was not analyzed. Third, there are many types of EBC collectors currently used internationally. Given that there is no uniform international standard, the results produced by different collection instruments are not comparable in an analysis. There is a need to standardize EBC collectors and international operating practices to attain repeatability and comparability of various research data. Additionally, a more comprehensive study of the COPD pathophysiology requires a comparison of MMP-12 and TIMP-4 levels in EBC with independent biomarkers of respiratory inflammation, including EBC biomolecules and electronic nose breathprints.

## Figures and Tables

**Table 1:** Clinical characteristics and demographics of the study participants.

Parameters	Healthy controls (*n* = 28)	COPD patients (*n* = 35)
Age (years)^†^	47.3 ± 15.8	70.9 ± 7.6
Sex (M/F)	17/11	23/12
Disease duration (years)	—	10.3 ± 5.5
Smoking history (nonsmoker/ex-smoker/current smoker)	21/7/0	3/25/7
Number taking (ICS/LABA/LAMA)	0/0/0	30/27/29
Pulmonary function		
FEV_1_ (% predicted)^†^	97 ± 24	49 ± 25
FVC (% predicted)^∗^	95 ± 23	71 ± 22
FEV_1_/FVC ratio (% predicted)^†^	87 ± 6	53 ± 14
DLco (% predicted)^†^	96 ± 21	55 ± 18

Parametric parameters were presented as mean ± SD. Differences between healthy controls and COPD patients (^†^*P* < 0.001 and ^∗^*P* = 0.006). *P* values were based on unpaired *t*-tests. *P* < 0.05 was considered significant. COPD: chronic obstructive pulmonary disease; ICS: inhaled corticosteroid; LABA: inhaled long-acting beta adrenergic antagonist; LAMA: inhaled long-acting antimuscarinic agonist; FEV_1_: forced expiratory volume in 1 second; DLCO: carbon monoxide diffusion capacity; FVC: forced vital capacity; PBMC: peripheral blood mononuclear cells; pred: predicted.

## Data Availability

The data used to support the findings of this study are available from the corresponding author upon request.
